# Duplex dPCR System for Rapid Identification of Gram-Negative Pathogens in the Blood of Patients with Bloodstream Infection: A Culture-Independent Approach

**DOI:** 10.4014/jmb.2103.03044

**Published:** 2021-09-11

**Authors:** Juyoun Shin, Sun Shin, Seung-Hyun Jung, Chulmin Park, Sung-Yeon Cho, Dong-Gun Lee, Yeun-Jun Chung

**Affiliations:** 1 Department of Microbiology, The Catholic University of Korea, College of Medicine, Seoul 06591, Republic of Korea; 2 Precision Medicine Research Center, Integrated Research Center for Genome Polymorphism, The Catholic University of Korea, College of Medicine, Seoul 06591, Republic of Korea; 3 Department of Biochemistry, The Catholic University of Korea, College of Medicine, Seoul 06591, Republic of Korea; 4 Vaccine Bio Research Institute, The Catholic University of Korea, College of Medicine, Seoul St. Mary’s Hospital, Seoul 06591, Republic of Korea; 5 Department of Internal Medicine, The Catholic University of Korea, College of Medicine, Seoul St. Mary’s Hospital, Seoul 06591, Republic of Korea

**Keywords:** Digital PCR, bloodstream infection, Gram-negative, blood-stream infection, antimicrobial resistance

## Abstract

Early and accurate detection of pathogens is important to improve clinical outcomes of bloodstream infections (BSI), especially in the case of drug-resistant pathogens. In this study, we aimed to develop a culture-independent digital PCR (dPCR) system for multiplex detection of major sepsiscausing gram-negative pathogens and antimicrobial resistance genes using plasma DNA from BSI patients. Our duplex dPCR system successfully detected nine targets (five bacteria-specific targets and four antimicrobial resistance genes) through five reactions within 3 hours. The minimum detection limit was 50 ag of bacterial DNA, suggesting that 1 CFU/ml of bacteria in the blood can be detected. To validate the clinical applicability, cell-free DNA samples from febrile patients were tested with our system and confirmed high consistency with conventional blood culture. This system can support early identification of some drug-resistant gram-negative pathogens, which can help improving treatment outcomes of BSI.

## Introduction

Bacterial bloodstream infection (BSI) is a life-threatening condition and a major healthcare issue across the world. Gram-negative bacteria are the most common organisms that cause severe BSI [1-4] and are responsible for high morbidity and mortality [5, 6]. Most BSI-causing gram-negative bacteria develop antimicrobial resistance [1, 7], hence, extended-spectrum β-lactamase (ESBL) producing bacteria and carbapenem-resistant Enterobacteriaceae are spreading rapidly [8]. Since inappropriate initial antibiotic therapy prolongs hospitalization and increases mortality rates, early and accurate detection of bacterial pathogens is important to improve clinical outcomes; especially, in the case of drug-resistant bacterial infections [9, 10].

Rapid identification of drug-resistant bacterial pathogens is essential to combat BSI. Blood culture is currently a gold standard for identifying bloodstream bacterial pathogens. With the introduction of automated blood culture systems, bacterial identification has become faster and more effective [11-13]. However, it still takes more than a day to identify bacteria and get final susceptibility results, which leads to either empiric use of unnecessary broad-spectrum antibiotics or delayed administration of appropriate antibiotics. Therefore, there is a need for rapid and sensitive bacterial detection systems. Multiplex identification and gram discrimination of clinically important drug-resistant bacterial pathogens are also important methods to control BSI. We have previously developed a novel tool for multiplex detection of antimicrobial resistant bacterial targets in the blood of BSI patients within 8-9 h, by integrating the principles of multiplex ligation-dependent probe amplification (MLPA) and capillary electrophoresis (CE) based single-strand conformation polymorphism (SSCP) [14-16]. Although this MLPA-CE-SSCP based system could achieve fast, multiplex bacterial identification, like other PCR-based tools [17-19], there is still room for improvement in sensitivity of the system for early diagnosis of BSI.

Recently, digital PCR (dPCR), the third generation PCR platform, has been developed. Its sensitivity is much higher than that of conventional PCR-based tools [20-22]. dPCR has been used to detect various pathogens and their antimicrobial resistance genes [23]. However, there are no multiplex dPCR systems for rapid identification of sepsis-causing gram-negative bacteria with antimicrobial resistance genes. Since the prevalence of drug-resistant gram-negative bacteria has increased, efficient identification of these bacteria is crucial for successful treatment of BSI.

In this study, we aimed to develop a dPCR system for culture-independent, multiplex, and highly sensitive detection of major sepsis-causing gram-negative bacteria and the antimicrobial resistance genes, and to validate our system using the plasma DNA of BSI patients.

## Materials and Methods 

### Bacterial Strains

The reference gram-negative bacterial strains and clinical isolates used in this study are listed in Supplemental Table 1. Five reference strains were obtained from the American Type Culture Collection (USA) and Korean Collection of Type Cultures (Korea). Thirteen clinical isolates were obtained from Seoul St Mary’s Hospital in South Korea [16].

### Digital PCR Primers and Probes

The species-specific primer/probe sets for gram-negative bacteria used in dPCR were prepared by modifying the sequences which have been verified by previous studies; *uidA* and *lacY* for *Escherichia coli* [24], *ompA* for *Acinetobacter baumannii* [25, 26], *phoE* for *Klebsiella pneumoniae* [27], and *ecfX* for *Pseudomonas aeruginosa* [28](Table 1). For detecting antimicrobial resistance genes, the primer/probe sets for TEM-type β-lactamase, CTX-M-1 group extended-spectrum β-lactamase, IMP metallo-β-lactamase, and New Delhi metallo-β-lactamase-1(NDM-1) were prepared by modifying previous data [29-31]. Specificity of the hybridization sequences of each probe was confirmed using BLAST (http://blast.ncbi.nlm.nih.gov/). Homology scores of the primer/probe sets for the resistance genes were calculated using the Clustal Omega software (http://www.ebi.ac.uk/Tools/msa/clustalo/).

### Digital PCR

dPCR experiments were performed using the QuantStudio 3D Digital PCR system (Thermo Fisher Scientific, USA) according to the manufacturer’s recommendations and the methodologies in a previous study [32]. A final volume of 14.5 μl sample which contained 7.2 μl of QuantStudio 3D Digital PCR Mastermix v2 (Thermo Fisher Scientific), 1 μl of genomic DNA, 0.75 μl of primer/probe set (final concentrations of 900 nM/250 nM, respectively), was loaded onto chips and sealed using the QuantStudio 3D Digital Chip Loader. PCR was carried out using the GeneAmp PCR system 9700 thermocycler (Applied Biosystems, USA), with the following cycling protocol: 96°C for 10 min, followed by 45 cycles of 60°C for 2 min and 98°C for 30 sec, and a final extension at 60°C for 2 min. After the amplification, the fluorescence signals were analyzed using the QuantStudio 3D Digital PCR software v3.0.

### Limit of Detection (LOD) and Limit of Blank (LOB) Test

To determine the LOD of our dPCR detection system, the genomic DNAs of *A. baumannii* ATCC 19606, *E. coli* ATCC 25922, *K. pnuemoniae* KCTC 12385, *P. aeruginosa* ATCC 27853, *bla*_TEM_ and *bla*_CTX-M-15_ encoding *E. coli* cm241, *bla*_NDM-1_ encoding *E. coli* cm66, and *bla*_IMP-1_ encoding *P. aeruginosa* cmPA-1 were serially diluted from 50 pg/ μl to 5 ag/μl and applied 1 μl of each DNA to the dPCR reaction. For LOB test, cell-free DNA (cfDNA) was extracted from plasma of normal human blood (Sigma-Aldrich, Germany) using the QIAamp Circulating Nucleic Acid Kit (Qiagen, Netherlands) and performed same dPCR using the blank cfDNA. LOB was defined as the number of positive signals measured in the cfDNA from normal blood as described elsewhere [33].

### Taqman qPCR

Quantitative polymerase reaction (qPCR) was carried out using the ViiA 7 QuantStudio real time PCR system (Thermo Fisher Scientific) with the following cycle protocol: initial denaturation at 95°C for 10 min, 45 cycles of amplification at 95°C for 10 sec, 60°C for 20 sec, and a 72°C for 20 sec. The amplicon mixture contained 5 μl of TaqMan Fast Advanced Master Mix (Applied Biosystems, USA), 0.5 μl of same primer/probe sets used for dPCR and 5 μl of cfDNA from patients’ blood. All qPCR reactions were performed in triplicate.

### Clinical Samples for dPCR

Thirty whole blood samples were collected from fifteen febrile patients (one from peripheral vein puncture and another one from central venous catheter of each patient) showing clinical signs of neutropenic fever: body temperature ≥38.0°C and an absolute neutrophil count of <500 cells/mm^3^ or a count of < 1,000 cells/mm^3^ with a predicted decrease to <500 cells/mm^3^ within the next two days as described elsewhere [34]. Seven blood samples that had no sign of BSI and negative blood culture results were also collected as negative controls. Five ml of the whole blood were centrifuged for 5 min at 1,200 g to isolate plasma. Cell-free DNA (cfDNA) from plasma was extracted using the QIAamp Circulating Nucleic Acid Kit (Qiagen). Same dPCR process described above was performed using the QuantStudio 3D Digital PCR system with the cfDNA samples. In parallel, blood culture was performed using sterile techniques. In addition to the blood culture, molecular diagnosis was also performed for each sample: 16s ribosomal RNA sequencing for bacteria identification and antimicrobial resistance gene PCR. Clinical sampling was performed at Seoul Saint Mary’s Hospital under the approval of Institutional Review Board of Catholic Medical Center (KC16TNSI0999). All procedures were performed in accordance with the relevant guidelines and regulations.

## Results

### Design and Validation of Primer and Probe Sets for dPCR

**Fig. 1:**
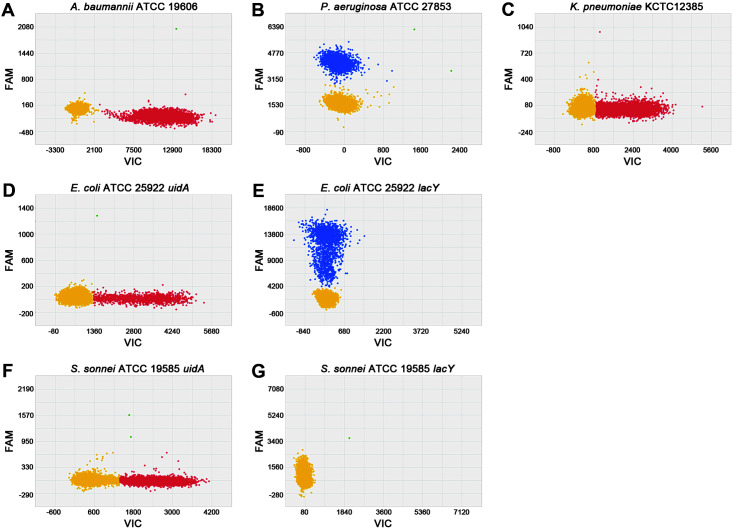
Scatterplots of target-specific singleplex digital PCR. (**A**) *A. baumannii* reference strain (ATCC 19606) detected specifically by the *ompA* primer-VIC probe set (x-axis, red). (**B**) *P. aeruginosa* reference strain (ATCC 27853) detected by the *ecfX* primer-FAM probe set (y-axis, blue). (**C**) *K. pneumoniae* reference strain (KCTC 12385) detected by the *phoE* primer-VIC probe set (x-axis, red). (**D** and **E**) *E. coli* reference strain (ATCC 25922) detected by the *uidA* primer-VIC (x-axis, red) and *lacY* primer-FAM (y-axis, blue) probe sets. (**F** and **G**) *S. sonnei* reference strain (ATCC 19585), was *uidA* positive but *lacY* negative. Yellow dots in the scatterplots represent no target-specific signal. X and Y-axis represent the intensities of VIC and FAM signals.

We designed five dPCR primer/probe sets targeting four major BSI-causing gram-negatives, *ompA* for *A. baumannii*; *ecfX* for *P. aeruginosa*; *phoE* for *K. pneumoniae*; *uidA* and *lacY* for *E. coli* (Table 1). Singleplex dPCR reactions were carried out with the reference strains to determine specificity of each primer/probe set (Fig. 1). All sets showed target-specific signals. ATCC 19606, the reference strain for *A. baumannii*, was specifically detected by the *ompA* primer-VIC probe set (Fig. 1A). ATCC 27853, the reference strain for *P. aeruginosa*, and KCTC 12385, the reference strain for *K. pneumoniae*, were specifically identified by the *ecfX* primer-FAM and *phoE* primer-VIC probe sets, respectively (Figs. 1B and 1C). ATCC 25922, the reference strain for *E. coli*, was specifically detected by both *uidA* primer-VIC and *lacY* primer-FAM probe sets (Figs. 1D and 1E). However, only *uidA* was detected in ATCC 19585, the reference strain for *S. sonnei*, (Figs. 1F and 1G). When the bacteria specific dPCR primer/probe sets were reacted with gram-positive bacterial DNA, no signal was observed (data not shown).

**Fig. 2:**
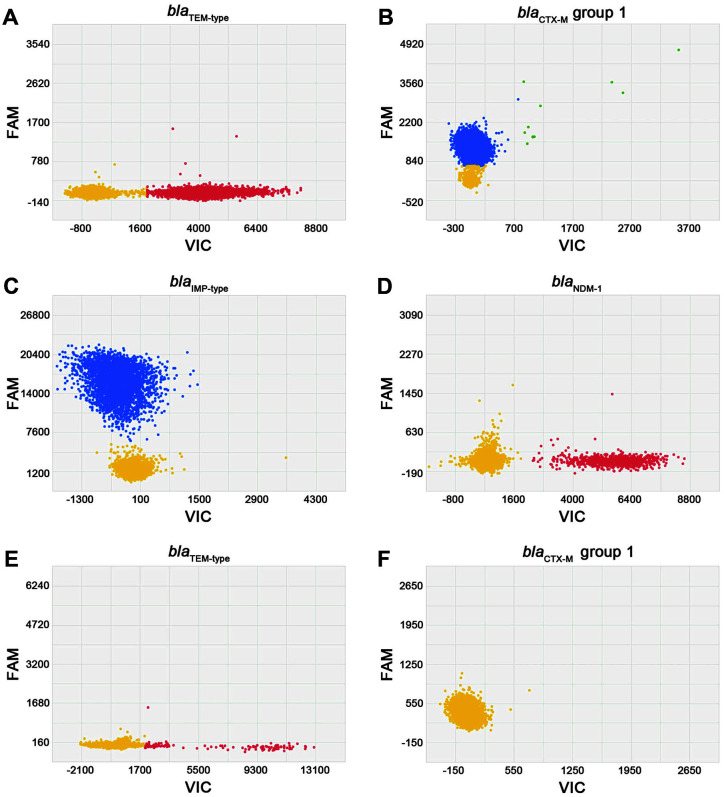
Target-specific identification of antimicrobial resistance genes by singleplex digital PCR. (**A** and **B**) The *bla*_TEM_ and *bla*_CTX-M-15_ genes detected specifically by the *bla*_TEM_ primer-VIC (x-axis, red) and *bla*_CTX-M_ group 1 primer-FAM probe sets (y-axis, blue) from the *E. coli* isolate, cm241. (**C**) The *bla*_IMP-1_ gene detected by the *bla*_IMP-1_ primer-FAM probe set (y-axis, blue) from *P. aeruginosa* isolate cmPA-1. (**D**) The *bla*_NDM-1_ detected by the *bla*_NDM-1_ primer-VIC probe set (x-axis, red) from the *E. coli* isolate, cmEC-1. (**E** and **F**) In the *E. coli* isolate cm66, *bla*_TEM_ gene was detected, but the *bla*_CTX-M_ group 1 gene was not.

In addition, we also tried to identify the presence of antimicrobial resistance genes in these pathogens. For this, we designed the dPCR sets targeting four major drug resistance genes; two ESBL genes, *bla*_TEM_ and *bla*_CTX-M_ group 1, and two metallo-beta-lactamase (MBL) genes, *bla*_IMP_ and *bla*_NDM-1_ (Table 1). To test the performance of the primer/probe sets, we analyzed 13 clinical isolates that were previously evaluated to encode the resistance genes (Table S1). We confirmed that the two ESBL genes were successfully detected in *E. coli* cm241 and *K. pneumoniae* 13B-333 (Figs. 2A and 2B). Both the MBL genes, *bla*_IMP_ and *bla*_NDM-1_ were specifically detected in *P. aeruginosa* isolates cmPA-1 and *E. coli* isolate cmEC-1, respectively (Figs. 2C and 2D). *E. coli* isolate cm66, known to produce CTX-M group 9 ESBL and *bla*_TEM_ enzymes [16], showed signal for *bla*_TEM_, but *bla*_CTX-M_ group 1 signal was not detected (Figs. 2 E and 2F).

### Detection Sensitivity and Specificity

To examine the sensitivity of our dPCR detection system, the genomic DNA of *A. baumannii* 19606 was serially diluted from 50 pg/μl (291.03 copies/μl) to 5 ag/μl (0.125 copies/μl), and applied to the dPCR system. The LOD was determined to be 50 ag (1.061 copies/μl) (Fig. S1). To compare detection sensitivity of our dPCR system with that of the TaqMan qPCR assay, which is the most commonly used PCR based technology for bacterial detection, we used the same reaction conditions. In TaqMan qPCR, the LOD of *A. baumannii* DNA was 500 fg (9.031 copies/μl)(Fig. S1), indicating that sensitivity of the dPCR assay was approximately 10^4^ times higher than that of the TaqMan qPCR assay. The LOD of all the other targets was also 50 ag except for *bla*_IMP-type_ (500 ag) (Fig. S2).

To determine the LOB for each target, we performed dPCR reactions using cfDNA extracted from normal blood in triplicates. LOBs for each target (on average) are as follows: 2 for *A. baumannii* (range 1-3), 2 for *P. aeruginosa*, 4.7 for *K. pneumoniae* (range 2-7), 3.7 for *E. coli* *uidA* gene (range 3-4), 3.7 for *E. coli* *lacY* gene (range 0-10), 1.3 for *bla*_TEM-type_ (range 0-3), 2 for *bla*_CTX-M_ group-1, 4.7 for *bla*_IMP-type_ (range 3-6), 1.3 for blaNDM (range 1-2), respectively (Fig S3). When we counted the number of positive signals in LOD of the nine dPCR targets, the average number was 44.7 (range 7-240), suggesting that the dPCR results are not false positive (Figs. S1 and S2).

### Duplex Identification of Gram-Negative Pathogens

**Fig. 3:**
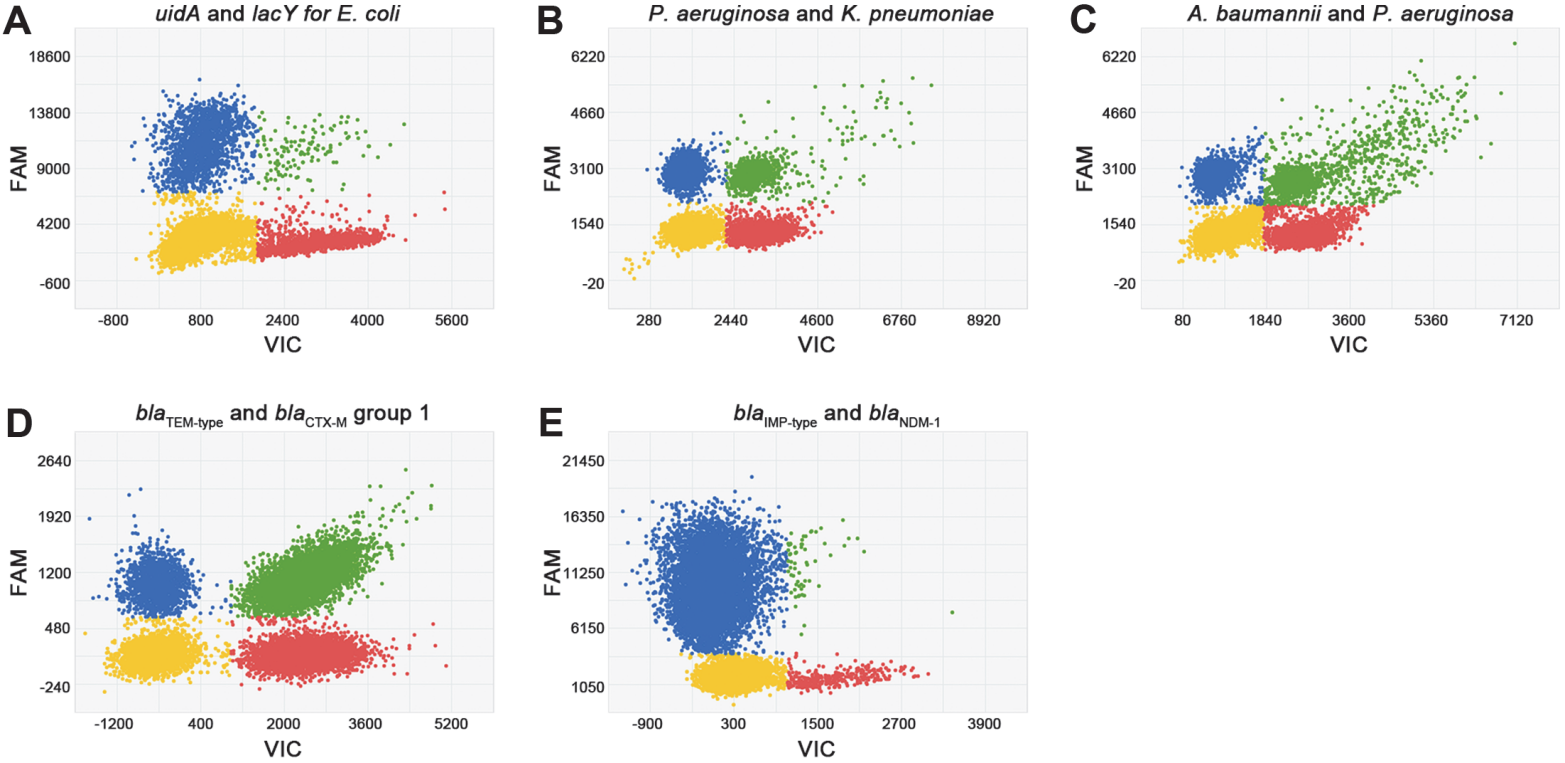
Duplex identification of target genes by digital PCR. (**A**) The *uidA* (x-axis, red) and *lacY* (y-axis, blue) genes specifically identified in a duplex reaction condition from the *E. coli* reference strain (ATCC 25922). (**B**) The *ecfX* (y-axis, blue) and *phoE* (x-axis, red) genes identified from the DNA mixture of *P. aeruginosa* and *K. pneumoniae*. (**C**) The *ompA* (x-axis, red) and *ecfX* (y-axis, blue) genes identified from the DNA mixture of *A. baumannii* and *P. aeruginosa*. (**D**) The *bla*_TEM-type_ (x-axis, red) and *bla*_CTX-M_ group 1 (y-axis, blue) genes identified from the *E. coli* isolate cm241. (**E**) The *bla*_IMP-type_ (y-axis, blue) and *bla*_NDM-1_ (x-axis, red) genes identified from the DNA mixture of *P. aeruginosa* cmPA-1 and *E. coli* cmEC-1 isolates.

After confirming the performance of the primer/probe sets using singleplex dPCR system, we tested whether the pathogens can be specifically detected under the duplex reaction conditions without cross-reactivity or interference between the different primer/probe sets. For duplex dPCR, we mixed the target-specific primer/probe sets with two different dyes (FAM and VIC dyes) and performed dPCR using 25 pg of each bacterial genomic DNA mixture per reaction. *uidA* primer-VIC and *lacY* primer-FAM probe sets clearly detected both *uidA* and *lacY* genes from *E. coli* (Fig. 3A). For duplex identification of *P. aeruginosa* and *K. pneumoniae*, we used the *ecfX* primer-FAM and *phoE* primer-VIC probe sets, respectively, and they specifically detected the *ecfX* and *phoE* genes (Fig. 3B). For identification of *A. baumannii* and *P. aeruginosa*, we used the *ompA* primer-VIC and *ecfX* primer-FAM probe sets, and they detected the *ompA* and *ecfX* genes, respectively (Fig. 3C). We observed no interference in any of the duplex dPCR experiments. To exclude non-specific amplification, we checked cross reactivity between each primer-probe set and unmatched gram-negative bacterial DNA. No non-specific signals were observed in any combination (data not shown). Next, we tried duplex identification of the four drug resistance genes. In duplex dPCR with combination of the *bla*_TEM-type_ primer-VIC and *bla*_CTX-M_ group 1 primer–FAM probes, both target specific signals were successfully detected from *E. coli* cm241 DNA (Fig. 3D). In another duplex dPCR with the combination of *bla*_IMP-type_ primer–VIC and *bla*_NDM-1_ primer–FAM probes, IMP and NDM-1 signals were successfully detected from the DNA mixture of *P. aeruginosa* cmPA-1 and *E. coli* cmEC-1, respectively (Fig. 3E). Taken together, the nine targets (five gram-negative bacteria specific targets and four antimicrobial resistance genes) were successfully identified by five duplex reactions. To minimize the time for the whole procedure, from blood sampling to pathogen identification, we unified the duplex dPCR conditions as described in Materials and Methods. Therefore, all five duplex dPCR reactions could be performed simultaneously, and the whole procedure took less than 3 hours.

### Detection of Gram-Negative Pathogens from BSI Patients’ Blood

The final goal of our study was to develop a sensitive and rapid culture-independent method for detection of major gram-negative pathogenic bacteria and drug resistance genes from blood of BSI patients. To accomplish this, we tested our duplex dPCR system using thirty blood samples collected from fifteen febrile patients: two blood samples were collected from each patient (central and peripheral vessels). In total, thirty cfDNA samples were used to test duplex dPCR system (five duplex reactions per sample). We found that 23 blood samples were culture positive (twenty for *E. coli*, two for *K. pnuemoniae*, and one for *P. aeruginosa*) (Table 2). All culture positive samples were consistently detected by the duplex dPCR. Of note, seven culture negative samples also showed bacteria specific signals in duplex dPCR (six *E. coli* and one *P. aeruginosa*) (Table 2). Examples of *E. coli* (*uidA* and *lacY* genes), *P. aeruginosa* (*ecfX*), and *K. pneumoniae* (*phoE*) identified from blood samples of three patients (18-C, 6-C and 36-C, respectively) by duplex dPCR are illustrated in Fig. S4. For comparison, TaqMan qPCR analyses were also performed for the same samples. In the TaqMan qPCR assay, 24 samples were bacteria positive (twenty-two for *E. coli* and two for *P. aeruginosa*), which were also consistently detected by duplex dPCR. However, of the six samples that were shown as bacteria negative by TaqMan qPCR, five were culture positive, suggesting that detection sensitivity of the TaqMan qPCR assay might be lower than that of the culture assay (Table 2).

Eight blood samples from febrile patients were detected with the antimicrobial resistance genes by the duplex dPCR (Table 2). Of the eight samples, seven were consistently identified by the culture and TaqMan qPCR. The genes in the sample 22-P were not detected either by culture or by TaqMan qPCR, but were detected by dPCR. Fig. S4 shows the examples of antimicrobial resistance genes identified from patient blood samples of three patients. When we examined the seven blood samples that had negative blood culture as negative controls, none of them showed bacterial pathogen specific signals by duplex dPCR, TaqMan qPCR or culture assay (data not shown).

## Discussion

Rapid identification of pathogens is essential to ensure appropriate antibiotic therapy and to improve clinical outcomes of BSI, especially in the case of drug-resistant bacterial infection [5-7, 9, 10]. Since the conventional blood culture takes 18-24 hours, it causes delay in administration of accurate antibiotics. PCR based technologies for detecting bacterial DNA, such as SepsiTest (Molzym, Germany) and SeptiFast (Roche, Switzerland) have also been developed. Although these assays have enabled faster detection of bacterial pathogens in patient’s blood, their sensitivity (28.6% for SepsiTest and 49.2% for SeptiFast) and specificity (88.2% and 85.3%, respectively) are not of clinical standards [18]. Polymerase chain reaction followed by electrospray ionization-mass spectrometry also showed a sensitivity of 81% and specificity of 69% at 6 hours sample acquisition [35]. In this study, we aimed to develop a culture-independent method, which can identify causal bacterial DNA from patient’s plasma cfDNA within 3-4 hours after sampling. Plasma of BSI patient contains DNAs released from the lysed bacterial cells and intact bacteria in addition to the DNAs from the lysed host cells, which would be useful resource for culture-independent pathogen identification. Although certain amount of pathogen DNA can be existed in the DNAs extracted from white blood cells due to their engulfment of pathogens [36, 37], abundant amount of human white blood cell DNA may hinder the detection of pathogen DNA. Therefore, we used plasma cfDNA for this development.

To improve sensitivity, we adopted a dPCR technique for identifying bacterial DNA and antimicrobial resistance genes. We designed dPCR primer/probe sets targeting major sepsis-causing gram-negative pathogens. For *E. coli*, we designed a probe targeting the *uidA* gene, which encodes β-glucuronidase, a universal target for *E. coli* detection. However, due to the genetic similarity between *E. coli* and *Shigella* spp., the *uidA* alone cannot distinguish *E. coli* from *Shigella* spp [24, 38]. Therefore, we designed an additional probe targeting *lacY*, an *E. coli*-specific gene encoding lactose permease. For *P. aeruginosa*, since the *ecfX* gene has proved to be an appropriate *P. aeruginosa*-specific target [28, 39], we selected this gene. For *K. pneumoniae*, we selected the *phoE* gene, which has been reported to be *K. pneumoniae*-specific [21]. For *A. baumannii*, the *ompA* gene was selected because of its specificity and sensitivity for detecting *A. baumannii* by real-time PCR [17, 20]. We also designed the dPCR primer/probe sets targeting four major drug resistance genes (*bla*_TEM-type_, *bla*_CTX-M_ group 1, *bla*_IMP-type_, and *bla*_NDM-1_). We confirmed the efficacy of all nine target genes using previously verified clinical isolates, indicating the accuracy of our system. The minimum detection limit of bacterial DNA by dPCR was 50 ag, which suggests that dPCR is 10^4^ times more sensitive than qPCR (500 fg). In principle, the amount of genomic DNA in one *E. coli* cell is approximately 0.017 pg, suggesting that dPCR can detect 1 CFU/ml of bacteria in the blood. Given that 1-100 CFU/ml of bacteria exist in the blood of adult BSI patients [40, 41], our dPCR system is sensitive enough to detect causal bacterial pathogens at an early stage of BSI.

The whole procedure of dPCR, from blood sampling to dPCR, took less than 3 hours, which fulfils the aim of this study. To avoid the total reaction time for multiple targets, we established a universal reaction condition for all the nine targets. In addition, we developed a duplex identification system for more efficient reaction, by mixing the target specific primer/probe sets with two different dyes, which enabled the detection of all nine targets by five duplex dPCR reactions.

To examine the clinical applicability of this system, thirty cfDNA samples were tested with our duplex dPCR system and the results were compared with those from blood cultures. All blood culture results were consistent with those from our duplex dPCR system, suggesting high detection sensitivity of our system. Interestingly, seven culture negative samples (five patients) showed positive pathogen signals in duplex dPCR. It may be due to that the pathogens could have been inactivated by antibiotic treatment before the blood was sampled. In that case, although blood culture was negative, small amount of bacterial DNA that was released in the blood from the destroyed pathogens could be detected by dPCR. Indeed, four of the five (80%) discordant patients were sampled after the initiation of antibiotic treatment, while eight of the ten (80%) concordant patients were sampled before antibiotic treatment in this study (Table S2). These results suggest that dPCR would be more sensitive than blood culture, a gold standard for identifying bloodstream infection. However, these results also indicate that ‘dPCR positive’ does not always mean the existence of live pathogen in the blood, which physicians must aware to interpret the result. One discordant case (117-C) was sampled before antibiotic treatment (Table S2), suggests that blood culture alone may miss positive infection. Taken together, combination of blood culture and molecular method such as dPCR can improve the sensitivity and reliability of diagnosis. All negative control samples showed negative results in dPCR assay, suggesting specificity of our dPCR system.

We further compared the performance of duplex dPCR with that of TaqMan qPCR, the most commonly used PCR-based technique for bacteria detection. All the pathogens and antimicrobial resistance genes identified by TaqMan qPCR were consistently detected by our duplex dPCR assay. Six samples identified as bacteria-negative by TaqMan qPCR were defined as bacteria-positive by dPCR and five of them were positive in blood cultures suggesting that the inconsistencies would be due to higher detection sensitivity of dPCR than qPCR assay. These results are compatible with a previous report [18]. The LODs of the dPCRs developed in this study were much higher than the LOBs of the same targets, further supporting that the dPCR results of the patients would not be false positives.

There are several limitations in this study. First, two ESBL and two MBL genes, the antimicrobial resistance genes targeted in this study, cannot cover other important ESBL and MBL genes such as KPC gene, VIM gene, and various CTX-M subtype genes. Second, our system does not cover gram-positive pathogens and related antimicrobial resistance genes. There is no doubt that gram-positive pathogens are critically important pathogens of BSI. We plan to expand the targets of this duplex dPCR system to major pathogens across gram-positives and negatives, and also to expand the target genes for covering more clinically important antimicrobial resistance genes. Third, the number of clinical isolates, blood samples from BSI patients, and negative control samples might not large enough to support the performance of our system. Further larger scale validation will be required to verify the sensitivity, specificity and clinical utility of this technology.

We developed a duplex dPCR based, culture-independent technique for identifying major BSI-causing gram-negative pathogens, and antimicrobial resistance genes in the cfDNA isolated from plasma of the patients. Our dPCR system detected nine targets (five bacteria-specific targets and four antimicrobial resistance genes) within 3 hours by five duplex dPCR reactions under universal condition. The duplex dPCR showed higher sensitivity than the TaqMan qPCR assays and blood cultures. To the best of our knowledge, this is the first dPCR system for screening major BSI-causing gram-negative bacteria, and major antimicrobial resistance genes together. This system can support early identification of some drug-resistant gram-negative pathogens, which can help improving treatment outcomes.

## Supplemental Materials

Supplementary data for this paper are available on-line only at http://jmb.or.kr.

## Figures and Tables

**Table 1:** Primer/probe sequences of targeted genes for dPCR.

	Target gene	Probe	Primer/probe sequences
*A. baumanii*	*ompA*	VIC	F: ACGTAGTTCTTGGTGGTCACTTGA R: AGGTCTTCAGTTAACTCTTGTGGTTGT P: CTCCTGTAGTAGAAGTTG
*E. coli*	*uidA*	VIC	F: GGGCGAACAGTTCCTGATCA R: TCATGACGACCAAAGCCAGTAA P: CCACAAACCGTTCTAC
	*lacY*	FAM	F: CTGGTCTGTTTCTGCTTCTTTAAGC R: TGCCCGCCAGTACAGACA P: ACTGGCGATGATTTT
*K. pneumoniae*	*phoE*	VIC	F: TGCAGTACCAGGGTAAAAACGA R: CGCCGTCGCCGTTCT P: CCGTGAAGCGAAGAA
*P. aeruginosa*	*ecf*	FAM	F: CCGCGCGCATTTCTTTT R: CCAATGGTCGCGCAACA P: CAGATCGCCCGCAAC
Resistance genes	*bla*_TEM-type_	VIC	F: TGCTGCCATAACCATGAGTGA R: GGTCCTCCGATCGTTGTCA P: TGCTGCCAACTTACT
	*bla*_CTX-M_ group 1	FAM	F: GACGCTGGGTAAAGCATTGG R: GGTATTGCCTTTCATCCATGTCA P: ACAGCCAACGGGC
	*bla*_NDM-1_	VIC	F: GACCGCCCAGATCCTCAA R: CGCGACCGGCAGGTT P: TGGATCAAGCAGGAGAT
	*bla*_IMP-type_	FAM	F: CGATCTATCCCCACGTATGCA R: GGCTTGAACCTTACCGTCTTTTT P: CTGAATTAACAAATGAACTGC

The Gibbs free energy (ΔG) of each probe sequence was determined using the Mfold software (http://mfold.rna.albany.edu/?q=mfold/); the probes with ΔG ≥ 0 were used in this study.

**Table 2:** Bacterial identification results by duplex dPCR, TaqMan PCR and blood culture

Blood sample		dPCR	TaqMan PCR	Blood culture
6-C	*ecfX*	*bla*_IMP-type_	*ecfX*, *bla*_IMP-type_	Negative
6-P	*ecfX*	*bla*_IMP-type_	*ecfX*, *bla*_IMP-type_	*P. aeruginosa* , *bla*_IMP-type_ positive
18-C	*uidA*, *lacY*	*bla*_TEM-type_, *bla*_CTX-M_ group 1	*uidA*, *lacY*, *bla*_TEM-type_, *bla*_CTX-M_ group 1	*E. coli* , *bla*_TEM-type_, *bla*_CTX-M_ group 1 positive
18-P	*uidA*, *lacY*	*bla*_TEM-type_, *bla*_CTX-M_ group 1	*uidA*, *lacY*, *bla*_TEM-type_, *bla*_CTX-M_ group 1	*E. coli* , *bla*_TEM-type_, *bla*_CTX-M_ group 1 positive
22-C	*uidA*, *lacY*	*bla*_TEM-type_, *bla*_CTX-M_ group 1	-	*E. coli* , *bla*_TEM-type_, *bla*_CTX-M_ group 1 positive
22-P	*uidA*, *lacY*	*bla*_TEM-type_, *bla*_CTX-M_ group 1	-	Negative
36-C	*phoE*	-	-	*K. pneumoniae* positive
36-P	*phoE*	-	-	*K. pneumoniae* positive
67-C	*uidA*, *lacY*	-	*uidA*, *lacY*	Negative
67-P	*uidA*, *lacY*	-	*uidA*, *lacY*	Negative
78-C	*uidA*, *lacY*	-	*uidA*, *lacY*	*E. coli* positive
78-P	*uidA*, *lacY*	-	*uidA*, *lacY*	*E. coli* positive
93-C	*uidA*, *lacY*	-	-	*E. coli* positive
93-P	*uidA*, *lacY*	-	-	*E. coli* positive
96-C	*uidA*, *lacY*	-	*uidA*, *lacY*	*E. coli* positive
96-P	*uidA*, *lacY*	-	*uidA*, *lacY*	*E. coli* positive
100-C	*uidA*, *lacY*	-	*uidA*, *lacY*	Negative
100-P	*uidA*, *lacY*	-	*uidA*, *lacY*	Negative
102-C	*uidA*, *lacY*	-	*uidA*, *lacY*	*E. coli* positive
102-P	*uidA*, *lacY*	-	*uidA*, *lacY*	*E. coli* positive
107-C	*uidA*, *lacY*	-	*uidA*, *lacY*	*E. coli* positive
107-P	*uidA*, *lacY*	-	*uidA*, *lacY*	*E. coli* positive
109-C	*uidA*, *lacY*	*bla*_TEM-type_, *bla*_CTX-M_ group 1	*uidA*, *lacY*, *bla*_TEM-type_, *bla*_CTX-M_ group 1	*E. coli* , *bla*_TEM-type_, *bla*_CTX-M_ group 1 positive
109-P	*uidA*, *lacY*	*bla*_TEM-type_, *bla*_CTX-M_ group 1	*uidA*, *lacY*, *bla*_TEM-type_, *bla*_CTX-M_ group 1	*E. coli* , *bla*_TEM-type_, *bla*_CTX-M_ group 1 positive
116-C	*uidA*, *lacY*	-	*uidA*, *lacY*	*E. coli* positive
116-P	*uidA*, *lacY*	-	*uidA*, *lacY*	*E. coli* positive
117-C	*uidA*, *lacY*	-	*uidA*, *lacY*	*E. coli* positive
117-P	*uidA*, *lacY*	-	*uidA*, *lacY*	Negative
119-C	*uidA*, *lacY*	-	*uidA*, *lacY*	*E. coli* positive
119-P	*uidA*, *lacY*	-	*uidA*, *lacY*	*E. coli* positive

For blood culture, 8-10 ml of each blood was inoculated into aerobic and anaerobic bottles, and then immediately transported to the clinical laboratory.

C, blood samples collected from central vessels; P, blood samples collected from peripheral vessels
