# Supplementary material for: Duplex dPCR System for Rapid Identification of Gram-Negative Pathogens in the Blood of Patients with Bloodstream Infection: A Culture-Independent Approach
Source: J Microbiol Biotechnol. 2021 Sep 11;31(11):1481–9. doi: 10.4014/jmb.2103.03044 (PMC9705831; doi:10.4014/jmb.2103.03044)
Supplement: Supplementary file 1 [file jmb-31-11-1481-supple.pdf]

https://doi.org/10.4014/jmb.2103.03044

Supplementary Figures

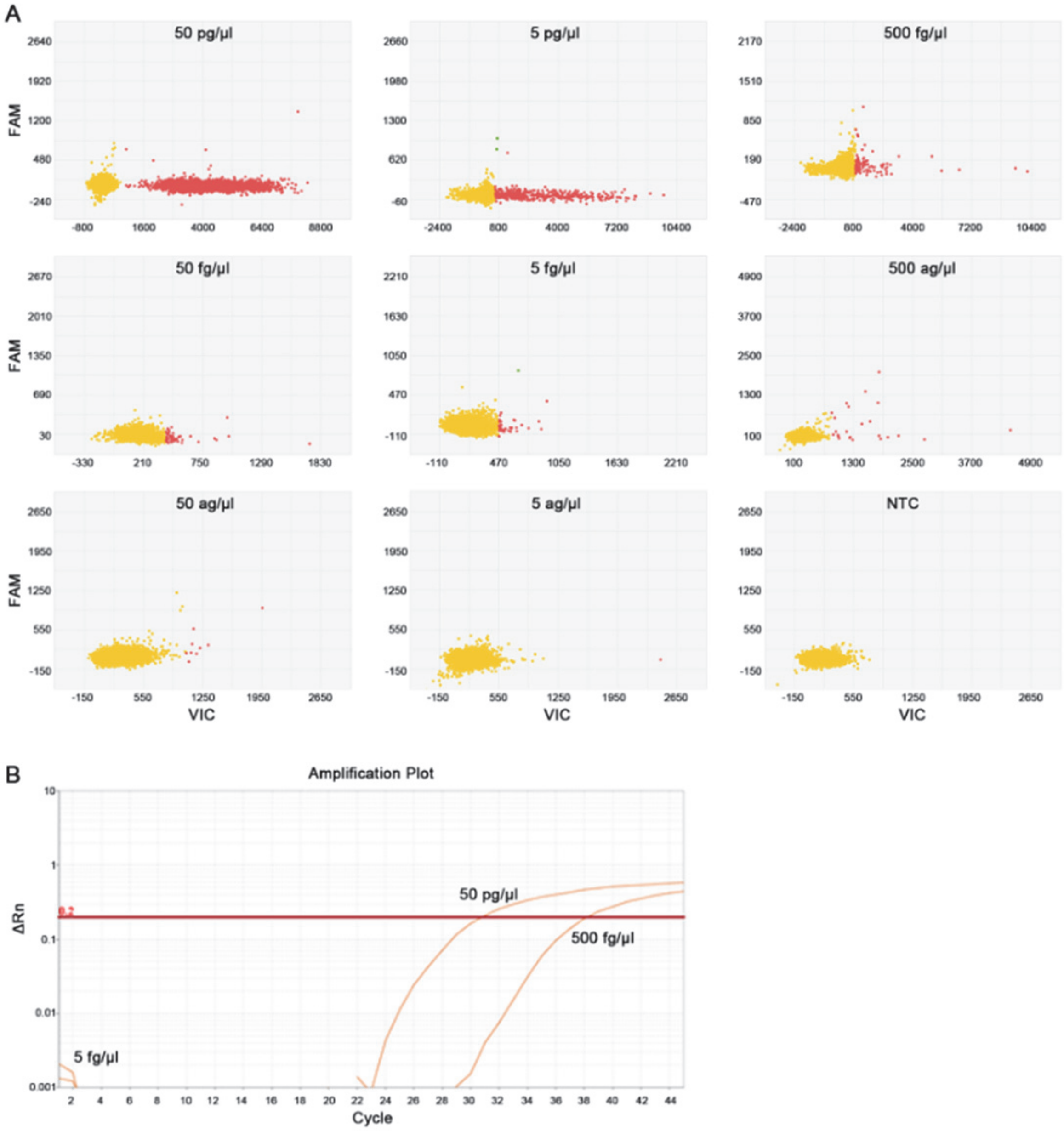

Figure S1. Limit of detection for *A. baumannii*. Genomic DNA of *A. baumannii* reference strain (ATCC 19606) was serially diluted from 50 pg/μl to 5 ag/μl and applied to both dPCR and TaqMan qPCR. (A) X-axis shows red fluorescence amplitude of the *A. baumannii* specific ompA primer-VIC probe signal by dPCR. (B) TaqMan qPCR amplification curves using 50 pg/μl, 500 fg/μl, 5 fg/μl of DNA isolated from *A. baumannii* (ATCC 19606).

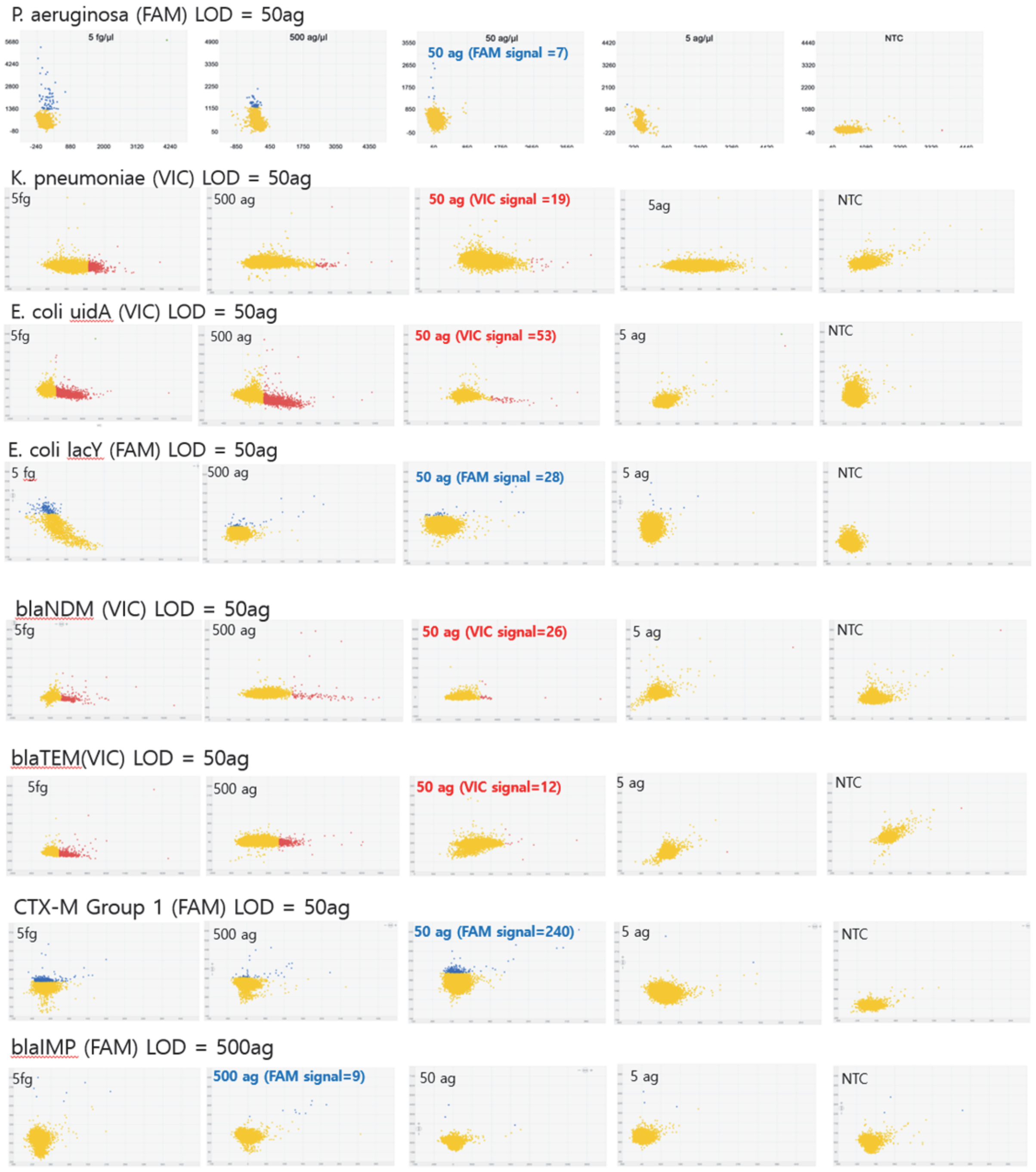

Figure S2. Limit of detection for eight pathogens. Genomic DNAs of *E. coli* ATCC 25922, *K. pnuemoniae* KCTC 12385, *P. aeruginosa* ATCC 27853, bla_{TEM} and $bla_{CTX\text{-}M\text{-}15}$ encoding *E. coli* cm241, $bla_{NDM\text{-}1}$ encoding *E. coli* cm66, and $bla_{IMP\text{-}1}$ encoding *P. aeruginosa* cmPA-1 were serially diluted from 50 pg/µl to 5 ag/µl and applied to both dPCR. The plots in this figure represent 5 fg/ul ~ 5 ag/ul. The LOD of all the targets was 50 ag except for $bla_{IMP\text{-}type}$ (500 ag). The numbers at each LOD point represent the number of positive dPCR signals.

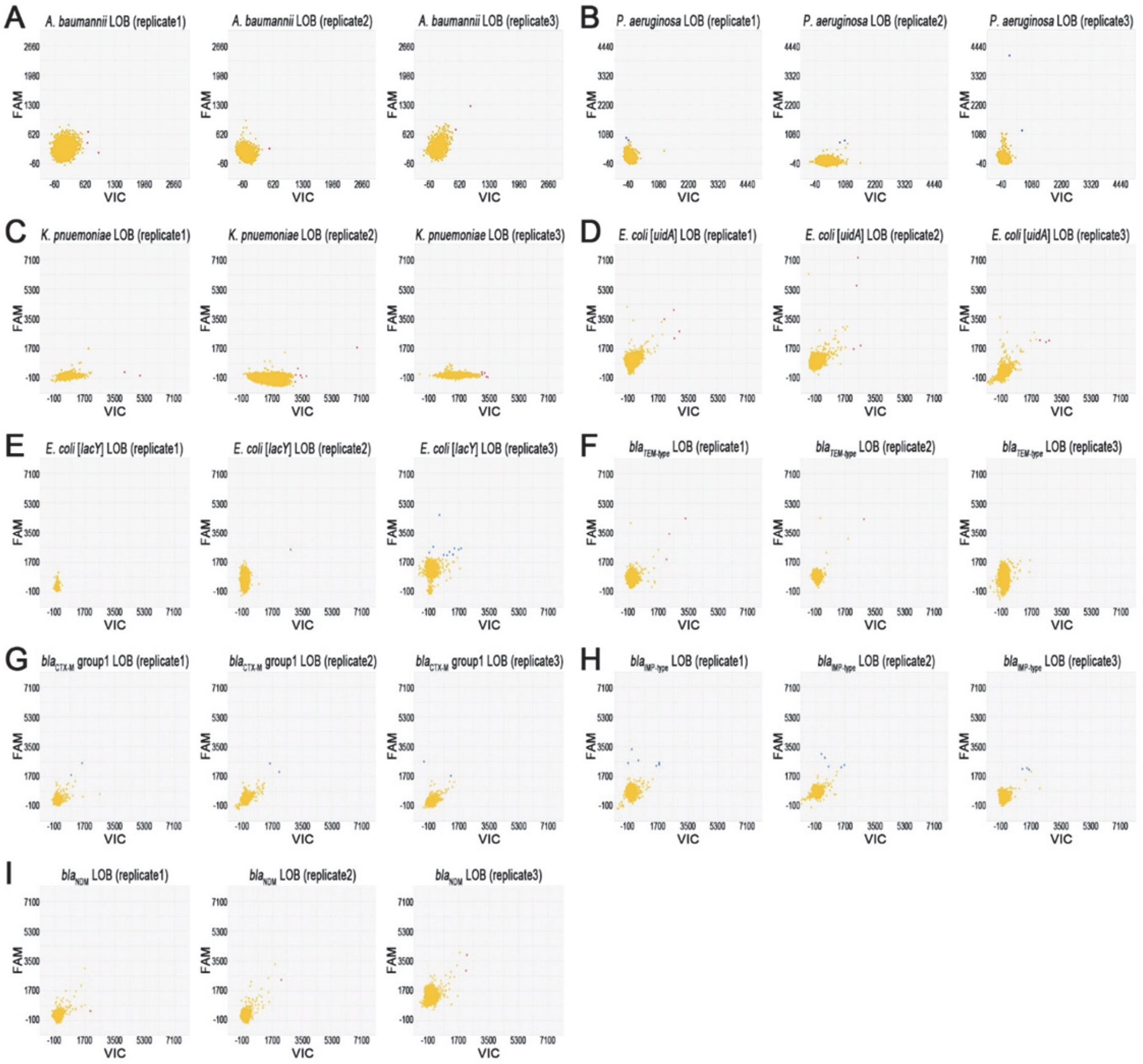

Figure S3. Limit of blank (LOB). The LOB evaluation of each target assay using cell-free DNA isolated from normal human blood. (A) *A. baumannii* assay (*ompA* gene) in triplicates (B) *P. aeruginosa* assay (*ecf* gene) in triplicates (C) *K. pneumoniae* assay (*phoE* gene) in triplicates (D) *E. coli* assay (*uidA* gene) in triplicates (E) *E. coli* assay (*lacY* gene) in triplicates (F) $bla_{\text{TEM-type}}$ gene assay in triplicates (G) $bla_{\text{CTX-M}}$ group 1 gene assay in triplicates (H) $bla_{\text{IMP-type}}$ gene assay in triplicates (I) bla_{NDM} gene assay in triplicates. LOB was defined as the number of positive signals.

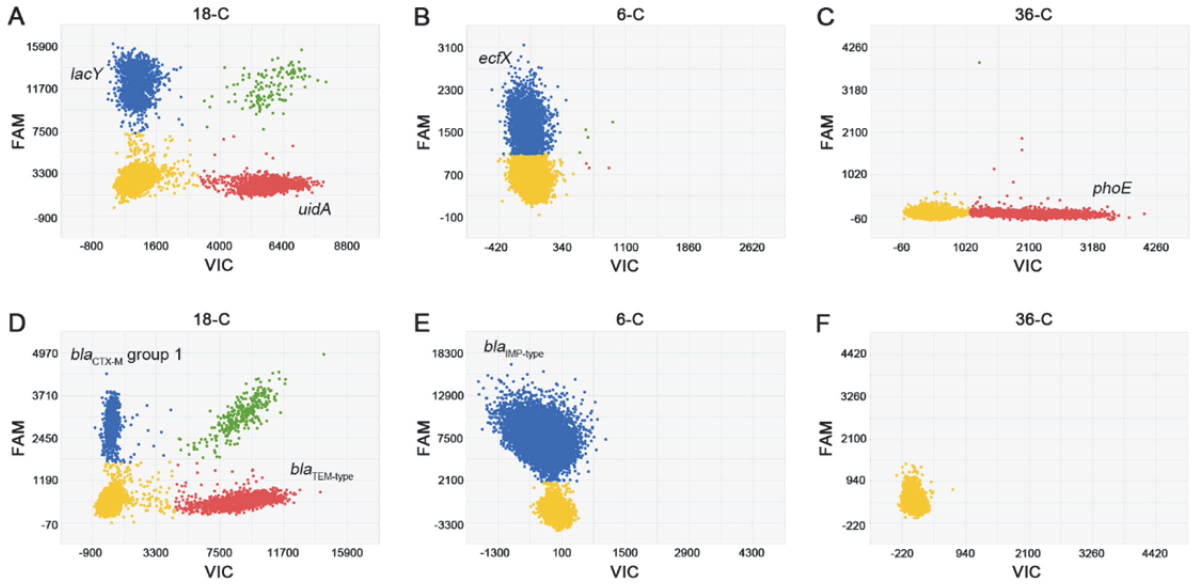

Figure S4. Detection of Gram-negative pathogens from sepsis patients' blood by duplex digital PCR. (A) *E. coli* specific genes, *uidA* (x-axis, red) and *lacY* (y-axis, blue), detected by duplex dPCR from the 18-C sample. (B) *P. aeruginosa* specific *ecfX* gene detected by the *ecfX* (y-axis, blue) and *phoE* (x-axis, red) duplex dPCR from the 6-C sample. (C) *K. pneumoniae* specific *phoE* gene detected by the *ecfX* (y-axis, blue) and *phoE* (x-axis, red) duplex dPCR from the 36-C sample. (D) The $bla_{\text{TEM-type}}$ (x-axis, red) and $bla_{\text{CTX-M}}$ group 1 (y-axis, blue) genes detected from the 18-C sample. (E) The $bla_{\text{IMP-type}}$ gene detected by the $bla_{\text{IMP-type}}$ (y-axis, blue) and $bla_{\text{NDM-1}}$ (x-axis, red) duplex dPCR from the 6-C sample. (F) No antimicrobial resistance gene was detected from the 36-C sample.

Supplementary Tables

Table S1. List of reference strains and clinical isolates

Strain	Species	Antimicrobial resistance gene[a]
ATCC 19606	*Acinetobacter baumannii*	
ATCC 25922	*Escherichia coli*	
KCTC 12385	*Klebsiella pneumoniae*	
ATCC 27853	*Pseudomonas aeruginosa*	
ATCC 19585	*Shigella sonnei*	
cm241[b]	*Escherichia coli*	bla_{TEM} , $bla_{\mathrm{CTX\text{-}M\text{-}15}}$
cm66[b]	*Escherichia coli*	bla_{TEM}, $bla_{\mathrm{CTX\text{-}M\text{-}9}}$
cmEC-1[b]	*Escherichia coli*	New Delhi metallo-β-lactamase-1 ($bla_{\mathrm{NDM\text{-}1}}$)
13B-333[b]	*Klebsiella pneumoniae*	bla_{TEM} , $bla_{\mathrm{CTX\text{-}M\text{-}15}}$
13B-236[b]	*Klebsiella pneumoniae*	
cmPA-1[b]	*Pseudomonas aeruginosa*	$bla_{\mathrm{IMP\text{-}1}}$
cmPA-2[b]	*Pseudomonas aeruginosa*	$bla_{\mathrm{IMP\text{-}1}}$
cmPA-4[b]	*Pseudomonas aeruginosa*	$bla_{\mathrm{IMP\text{-}1}}$
Aci_080004[b]	*Acinetobacter baumannii*	
Aci_080014[b]	*Acinetobacter baumannii*	
Aci_090023[b]	*Acinetobacter baumannii*	
Aci_090047[b]	*Acinetobacter baumannii*	
Aci_090050 [b]	*Acinetobacter baumannii*	

[a] Antimicrobial resistance genes were confirmed by PCR-sequencing
[b] The strains were obtained from Seoul St Mary's Hospital in South Korea.
Each strain was cultured anaerobically on a blood agar plate (Sigma-Aldrich, Darmstadt, Germany), and Luria-Bertani (LB) agar plate (BD, Franklin Lakes, NJ, USA) at 37 °C for 24 h.

Table S2. Point of blood sampling and phenotype of the strains isolated from the blood culture

Patient	Blood sample	Blood sampling	Resistant antibiotics
6	6-C	After	-
	6-P		MEM, PIP/TAZO, FEP, CIP
18	18-C	Before	CAZ, AZT, CIP
	18-P		CAZ, AZT, CIP
22	22-C	After	CAZ, AZT, CIP
	22-P		-
36	36-C	Before	-
	36-P		-
67	67-C	After	-
	67-P		-
78	78-C	Before	AMP
	78-P		AMP
93	93-C	Before	-
	93-P		-
96	96-C	Before	CAZ, AZT, CIP, TMP/SMX
	96-P		CAZ, AZT, CIP, TMP/SMX
100	100-C	After	-
	100-P		-
102	102-C	After	-
	102-P		-
107	107-C	Before	-
	107-P		-
109	109-C	After	CAZ, AZT, IMI (I)
	109-P		CAZ, AZT, IMI (I)
116	116-C	Before	-
	116-P		-
117	117-C	Before	-
	117-P		-
119	119-C	Before	-
	119-P		-

Of the fifteen patients, the blood sampling was performed before initiation of antibiotics treatment in nine cases. In six patients, the blood sampling was performed after antibiotics treatment. We excluded the patients whose neutropenic fever was persistent, but the peak of body temperature decreased. Antibiotic susceptibility testing was performed with an automatic system (Vitek-2, BioMeriux, Hazelwood, MO, USA).

C, blood samples collected from central vessels; P, blood samples collected from peripheral vessels

After, blood sampling after antibiotics treatment; Before, blood sampling before antibiotics treatment

AMP, ampicillin; AZT, aztreonam; CAZ, Ceftazidime; CIP, ciprofloxacin; FEP, cefepime; IMI, imipenem; MEM, meropenem; PIP/TAZO, piperacillin/tazobactam; TMP/SMX, trimethoprim/sulfamethoxazole; (I), intermediate resistance
